# Supplementary material for: A Systematic Review of the Prevalence of Persistent Gastrointestinal Symptoms and Incidence of New Gastrointestinal Illness after Acute SARS-CoV-2 Infection
Source: Viruses. 2023 Jul 26;15(8):1625. doi: 10.3390/v15081625 (PMC10459193; doi:10.3390/v15081625)
Supplement: Supplementary file 1 [file viruses-15-01625-s001.zip › viruses-2491088-supplementary.pdf]

Supplementary materials

Supplementary File S1. Search terms used for OVID MedLine, SCOPUS, Europe PubMed Central and medRxiv.

MedLine Search Terms

1. COVID-19.mp. or COVID-19/
2. SARS-CoV-2.mp. or SARS-CoV-2/
3. coronavirus disease 2019.mp. or COVID-19/
4. 1 or 2 or 3
5. Gastrointestinal Diseases/ or gastrointestinal symptoms.mp.
6. abdominal pain.mp. or Abdominal Pain/
7. Vomiting/ or vomiting.mp.
8. Diarrhea/ or diarrh?ea.mp. or Diarrhea, Infantile/
9. Constipation/ or constipation.mp.
10. malnutrition.mp. or Malnutrition/
11. gastritis.mp. or Gastritis/
12. Gastroesophageal Reflux/ or gord.mp.
13. reflux.mp. or Gastroesophageal Reflux/ or Bile Reflux/
14. pancreatitis.mp. or Pancreatitis/
15. Colitis/ or colitis.mp.
16. Esophagitis/ or Deglutition Disorders/ or oesophagitis.mp.
17. transaminitis.mp.
18. cholestasis.mp. or Cholestasis/
19. cholestatic liver injury.mp.
20. appendicitis.mp. or Appendicitis/
21. Gastrointestinal Hemorrhage/ or upper gastrointestinal bleed.mp.
22. Gastrointestinal Hemorrhage/ or lower gastrointestinal bleed.mp.
23. 5 or 6 or 7 or 8 or 9 or 10 or 11 or 12 or 13 or 14 or 15 or 16 or 17 or 18 or 19 or 20 or 21 or 22
24. Longitudinal Studies/ or longitudinal.mp.
25. observational.mp. or Observational Study/

26. Cross-Sectional Studies/ or cross-sectional.mp.
27. Cohort Studies/ or cohort.mp.
28. Case-Control Studies/ or case control.mp.
29. 24 or 25 or 26 or 27 or 28
30. 4 and 23 and 29
31. limit 30 to (english language and humans and yr="2019 -Current")

SCOPUS Search Terms

((TITLE-ABS (covid-19)) OR (TITLE-ABS (sars-cov-2)) OR (TITLE-ABS ("coronavirus disease 2019"))) AND ((TITLE-ABS ("gastrointestinal disease*")) OR (TITLE-ABS ("gastrointestinal symptom*")) OR (TITLE-ABS ("abdominal pain")) OR (TITLE-ABS (nausea)) OR (TITLE-ABS (vomiting)) OR (TITLE-ABS (diarrh*ea)) OR (TITLE-ABS (constipation)) OR (TITLE-ABS (malnutrition)) OR (TITLE-ABS (gastritis)) OR (TITLE-ABS (colitis)) OR (TITLE-ABS (*esophagitis)) OR (TITLE-ABS (transaminitis)) OR (TITLE-ABS (cholestasis)) OR (TITLE-ABS (appendicitis)) OR (TITLE-ABS ("gastrointestinal haemorrhage")) OR (TITLE-ABS ("upper gastrointestinal bleed")) OR (TITLE-ABS ("lower gastrointestinal bleed")) OR (TITLE-ABS ("cholestatic liver injury"))) AND ((TITLE-ABS (longitudinal)) OR (TITLE-ABS (cross-sectional)) OR (TITLE-ABS (cohort)) OR (TITLE-ABS ("case control")) OR (TITLE-ABS (case-control)) OR (TITLE-ABS (observational)))

Europe PMC Search Terms

((TITLE-ABS(covid-19)) OR (TITLE-ABS(sars-cov-2)) OR (TITLE-ABS("coronavirus disease 2019"))) AND ((TITLE-ABS("gastrointestinal disease*")) OR (TITLE-ABS("gastrointestinal symptom*")) OR (TITLE-ABS("abdominal pain")) OR (TITLE-ABS(nausea)) OR (TITLE-ABS(vomiting)) OR (TITLE-ABS (diarrh*ea)) OR (TITLE-ABS(constipation)) OR (TITLE-ABS(malnutrition)) OR (TITLE-ABS(gastritis)) OR (TITLE-ABS(colitis)) OR (TITLE-ABS(*esophagitis)) OR (TITLE-ABS(transaminitis)) OR (TITLE-ABS(cholestasis)) OR (TITLE-ABS(appendicitis)) OR (TITLE-ABS("gastrointestinal haemorrhage")) OR (TITLE-ABS ("upper gastrointestinal bleed")) OR (TITLE-ABS("lower gastrointestinal bleed")) OR (TITLE-ABS("cholestatic liver injury"))) AND ((TITLE-ABS(longitudinal)) OR (TITLE-ABS(cross-sectional)) OR (TITLE-ABS(cohort)) OR (TITLE-ABS("case control")) OR (TITLE-ABS(case-control)) OR (TITLE-ABS(observational)))

medRxiv (searched using the “medRxiv” API package in R studio)

```
topic1 <- c("covid-19","COVID-19","sars-[Cc][Oo][Vv]-2","SARS-[Cc][Oo][Vv]-2","coronavirus disease 2019")   # Combined with Boolean OR
topic2 <- c("gastrointestinal disease","gastrointestinal symptom*","abdominal pain","nausea","vomiting","diarr*ea","constipation","malnutrition","gastritis","colitis","*esophagitis","transaminitis","cholestasis","appendicitis","gastrointestinal haemorrhage","upper gastrointestinal bleed","lower gastrointestinal bleed","cholestatic liver injury")
```

```
topic3 <- c("longitudinal","cross-sectional","cross sectional","cohort","case control","case-control","observational") # Combined with Boolean OR
myquery <- list(topic1, topic2
```

Supplementary Figure S1. Funnel plot of study bias for five studies reporting an odds ratio for persistent diarrhoea. The funnel and pooled effect estimate shown in the dotted lines is based on a random effects model, though we do not interpret this further due to significant inter-study heterogeneity (see Main Text).

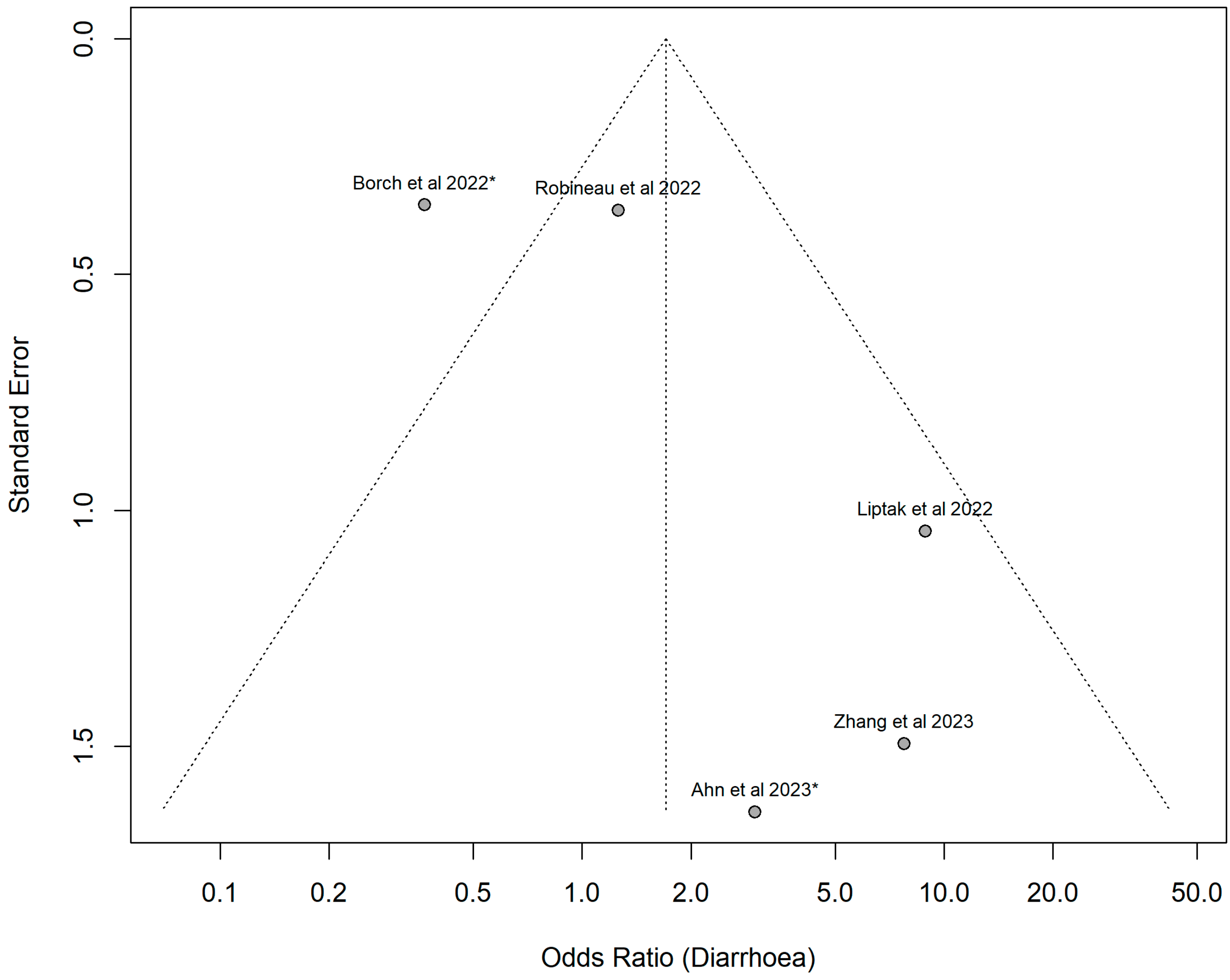

Supplementary Figure S2. Funnel plot of study bias for six studies reporting an odds ratio for persistent nausea and vomiting. The funnel and pooled effect estimate shown in the dotted lines is based on a random effects model, though we do not interpret this further due to significant inter-study heterogeneity (see Main Text).

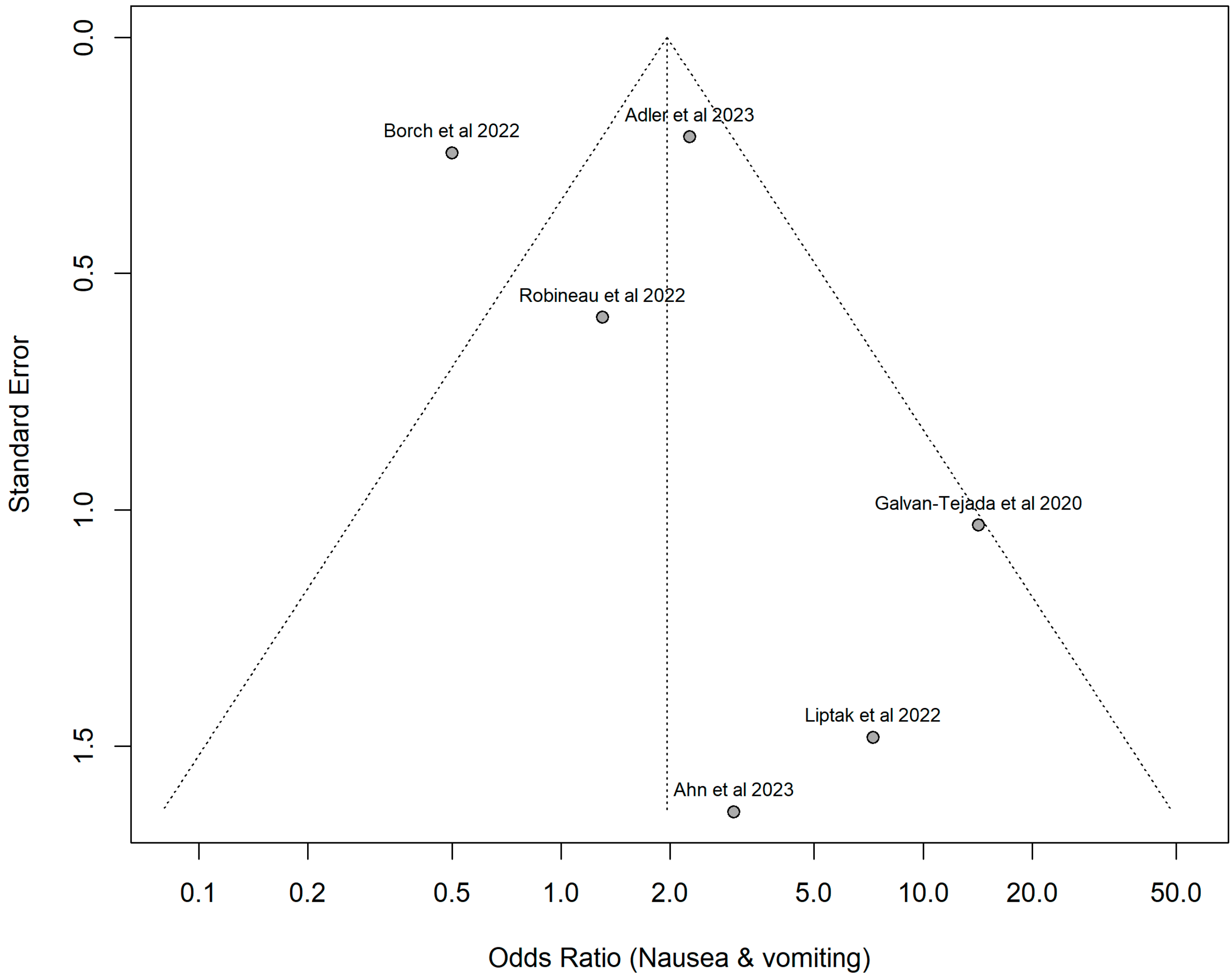

Supplementary Figure S3. Funnel plot of study bias for four studies reporting an odds ratio for persistent taste and smell disorders. The funnel and pooled effect estimate shown in the dotted lines is based on a random effects model, though we do not interpret this further due to significant inter-study heterogeneity (see Main Text).

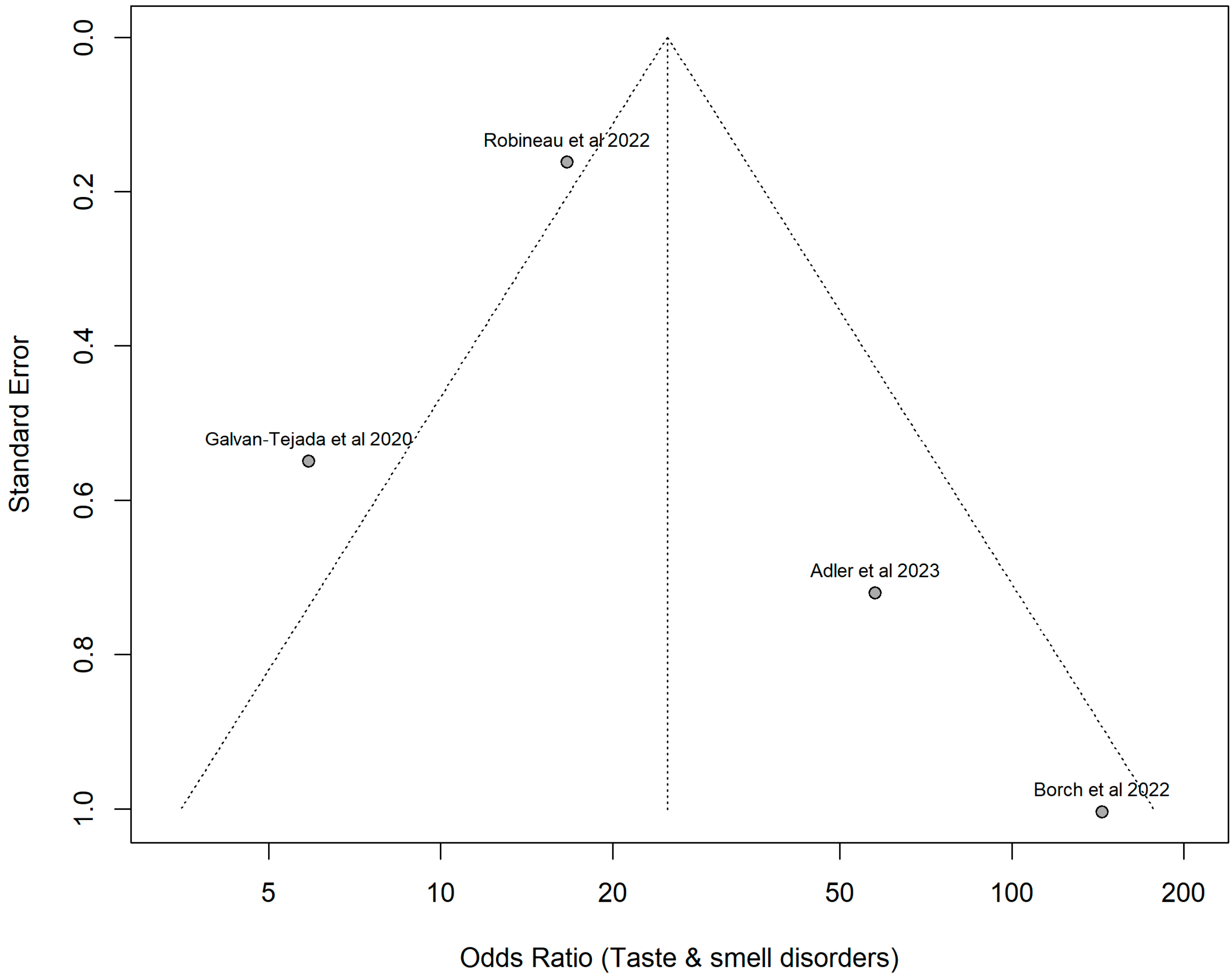

Supplementary Figure S4. Funnel plot of study bias for five studies reporting an odds ratio for persistent abdominal pain. The funnel and pooled effect estimate shown in the dotted lines is based on a random effects model, though we do not interpret this further due to significant inter-study heterogeneity (see Main Text).

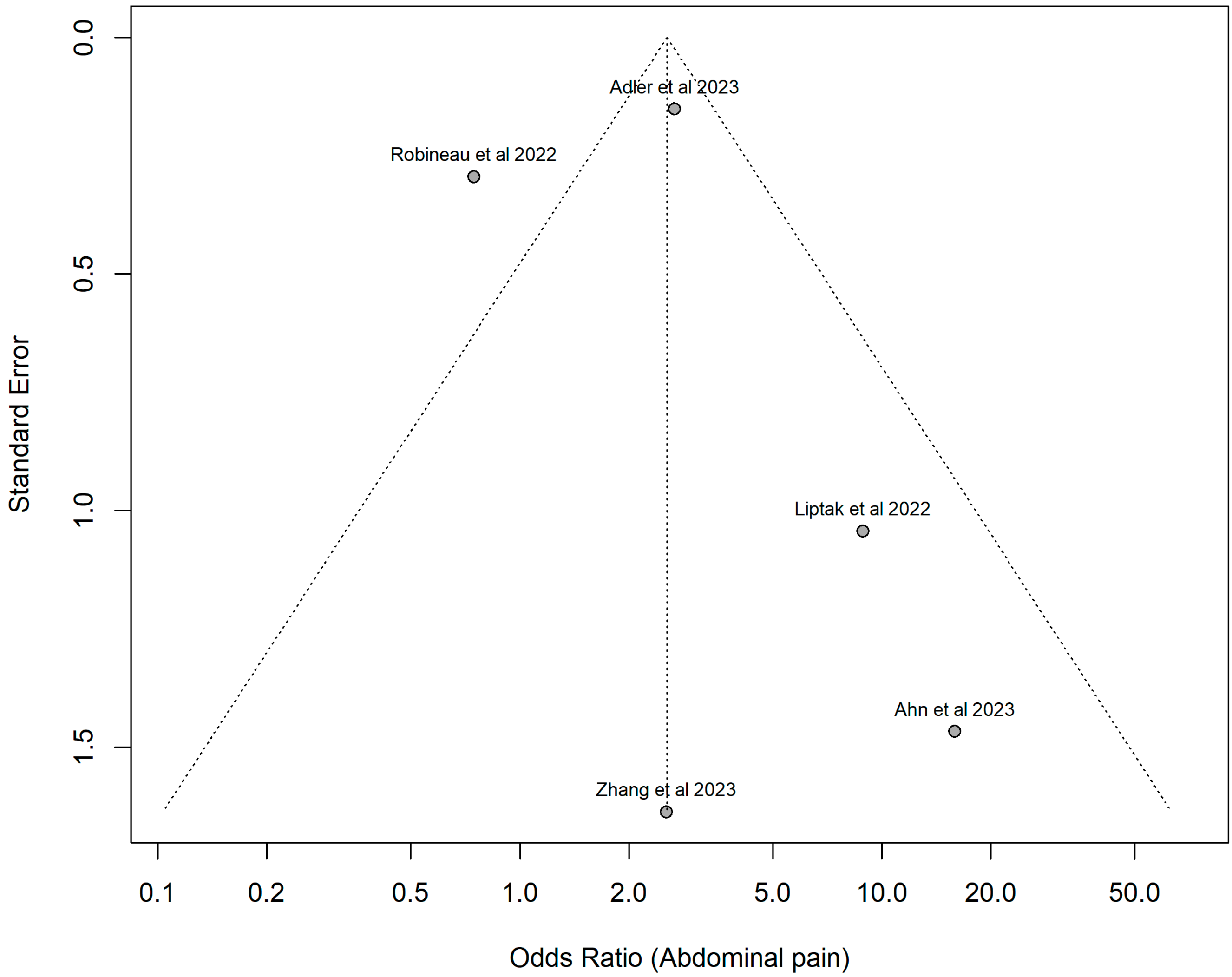

Supplementary Table S1. Risk of bias assessment, including justification for studies deemed to be of moderate or low quality.

**Author and year**	**Country**	**Overall quality of study**	**Reasoning for medium or high risk studies**
Islam, M. et al 2021[30]	Bangladesh	Moderate	Exposure not reliably measured, inclusion criteria not clearly defined.
Ghoshal, U.C. et al 2021[50]	Bangladesh and India	High	N/A

Liang, L. et al 2020[41]	China	Moderate	Absence of a comparator group, did not adjust for confounding variables.
Xie, XP. et al 2021[42]	China	Low	Absence of a comparator group, did not adjust for confounding variables, no information on participants lost to follow-up.
Attauabi, M. et al 2021[46]	Denmark	Low	Absence of a comparator group, did not adjust for confounding variables.
Borch, L. et al 2022[52]	Denmark	Moderate	Did not adjust for confounding variables.
Vaillant, MF. et al 2021[33]	France	Low	Absence of comparator group, participants potentially not free of outcome at study enrolment.
Faycal, A. et al. 2021[39]	France	Moderate	Absence of a comparator group.
Gerard, M. et al 2021[34]	France	Moderate	Absence of a comparator group, did not adjust for confounding variables, no information on participants lost to follow-up.
Belkacemi, M. et al 2022[37]	France	Low	Absence of comparator group, did not adjust for confounding factors, participants potentially not free of outcome at study enrolment.
Robineau, O. et al 2022[40]	France	High	N/A
Augustin, M. et al 2021[48]	Germany	Moderate	Absence of a comparator group.
Noviello, D. et al 2021[49]	Italy	High	N/A
Comelli, A. et al 2022[45]	Italy	Moderate	Absence of a comparator group, no information on participants lost to follow-up.
Damanti, S. et al 2022[35]	Italy	Low	Absence of a comparator group, exposure method not reliably measured, did not control for confounding variables, participants potentially not free of outcome at study enrolment, no information on participants lost to follow-up.
Fatima, G. et al 2021[71]	India	Low	Absence of comparator group, did not adjust for confounding variables, participants potentially not free of outcome at study enrolment.
Rao, G. et al 2021[70]	India	Low	Exposure method not reliably measured, did not adjust for confounding variables, study subjects and setting not clearly described.
Fernandez-Plata, R. et al 2022[53]	Mexico	Moderate	Absence of a comparator group, no information on participants lost to follow-up.
Galvan-Tejada, C. et al 2020[51]	Mexico	High	N/A
Qamar, M. et al 2022[32]	Pakistan	Moderate	Lack of information on outcome measurement.
Stepan, M. D. et al 2022[44]	Romania	High	N/A
Khodeir, M. et al 2021[31]	Saudi Arabia	Moderate	Inclusion criteria not clearly defined, did not adjust for confounding factors.

Liptak, P. et al 2022[47]	Slovakia	High	N/A
Karaarslan, F. et al 2021[36]	Turkey	Low	Absence of a comparator group, did not adjust for confounding factors.
Penner, J. et al 2021[43]	UK	Moderate	Absence of a comparator group, did not adjust for confounding factors.
Taquet, M. et al 2021[8]	USA	High	N/A
Wu, Q. et al 2022[38]	USA	High	N/A
Austhof, E. et al. 2022[54]	USA	Moderate	Absence of a comparator group, did not adjust for confounding factors.
Adler et al. 2023[55]	Israel	Moderate	Did not adjust for confounding factors.
Ahn et al. 2023[56]	South Korea	Moderate	Did not adjust for confounding factors, exposure not reliably measured.
Chancharoenthana et al. 2023[57]	Thailand	Moderate	Absence of a comparator group, did not adjust for confounding factors.
da Costa e Silva et al. 2023[58]	Brazil	Low	Absence of a comparator group, did not adjust for confounding factors, no information on participants lost to follow-up.
Dagher et al. 2023[59]	USA	Low	Absence of a comparator group, did not adfjust for confounding variables, participants potentially not free of outcome at study enrolment.
Fernandez-de-Las-Penas et al. 2023[60]	Spain	Moderate	Absence of a comparator group, did not adjust for confounding variables.
Fischer et al. 2022[61]	Luxembourg	Moderate	Absence of a comparator group.
Golla et al. 2023[62]	India	Moderate	Did not adjust for confounding factors, no information on participants lost to follow-up.
Imoto et al. 2022[63]	Japan	High	N/A
Karuna et al. 2023[64]	USA, Peru, Malawi, South Africa, Zambia and Zimbabwe	Moderate	Absence of a comparator group, participants potentially not free of outcome at study enrolment.
Sedik 2023[65]	Iraq	Low	Absence of a comparator group, did not adjust for confounding variables, participants potentially not free of outcome at study enrolment.
Xu et al. 2023[66]	USA	Moderate	Exposure not reliably measured, , no information on participants lost to follow-up.
Yamamoto et al. 2023[67]	Japan	Moderate	Absence of a comparator group.
Zhang et al. 2023[68]	China	High	N/A
Chang et al. 2023[69]	Not specified	High	N/A
Andersson et al. 2023[73]	Denmark	High	N/A

Ma et al. [72]	UK	High	N/A
